# Association of treatments for acute appendicitis with pregnancy outcomes in the United States from 2000 to 2016: Results from a multi-level analysis

**DOI:** 10.1371/journal.pone.0260991

**Published:** 2021-12-13

**Authors:** Jianzhou Yang, Shi Wu Wen, Daniel Krewski, Daniel J. Corsi, Mark Walker, Donald Mattison, Ryan Moog, Doug McNair, Huiping Huang, Guihua Zhuang

**Affiliations:** 1 Department of Epidemiology and Biostatistics, School of Public Health, Xi’an Jiaotong University Health Science Center, Xi’an, Shaanxi, China; 2 Department of Public Health and Preventive Medicine, Changzhi Medical College, Changzhi, Shanxi, China; 3 OMNI Research Group, Ottawa Hospital Research Institute, Ottawa, Ontario, Canada; 4 School of Epidemiology and Public Health, University of Ottawa, Ottawa, Ontario, Canada; 5 Department of Obstetrics and Gynecology, Faculty of Medicine, University of Ottawa, Ottawa, Ontario, Canada; 6 McLaughlin Centre for Population Health Risk Assessment, Faculty of Medicine, University of Ottawa, Ottawa, Ontario, Canada; 7 Risk Sciences International, Ottawa, Ontario, Canada; 8 Epidemiology and Biostatistics, Arnold School of Public Health, University of South Carolina, Columbia, SC, United States of America; 9 Cerner Corporation, Kansas City, Missouri, United States of America; 10 Bill & Melinda Gates Foundation, Seattle, Washington, United States of America; 11 Department of Infection Control, The First Affiliated Hospital of Xia Men University, Xiamen, Fujian, China; China Medical University, CHINA

## Abstract

**Background:**

Open appendectomy, laparoscopic appendectomy, and non-surgical treatment are three options to treat acute appendicitis during pregnancy. Previous studies on the association of different treatment methods for acute appendicitis with pregnancy outcomes have been limited by small sample sizes and residual confounding, especially with respect to hospital-level factors. This study aimed to investigate the association of treatment method for acute appendicitis with pregnancy outcomes using a multi-level analysis.

**Methods:**

A retrospective cohort study was conducted based on a large electronic health records database in the United States during the period 2000 to 2016. All pregnancies diagnosed with acute appendicitis and treated in participating hospitals during the study period were included. We conducted multi-level hierarchical logistic regression to analyze both individual- and hospital-level factors for abortion, preterm labor, and cesarean section.

**Results:**

A total of 10,271 acute appendicitis during pregnancy were identified during the study period. Of them, 5,872 (57.2%) were treated by laparoscopic appendectomy, 1,403 (13.7%) by open appendectomy, and 2,996 (29.2%) by non-surgical treatment. Compared with open appendectomy, both laparoscopic appendectomy (adjusted OR, 0.6, 95% CI, 0.4, 0.9) and non-surgical treatment (adjusted OR, 0.4; 95% CI, 0.3–0.7) showed a decreased risk of preterm labor. Other important individual-level determinants of adverse pregnancy outcomes included maternal age, gestational hypertension, and anemia during pregnancy, the hospital-level determinant included the number of beds.

**Conclusions:**

Compared with open appendectomy, both laparoscopic appendectomy and non-surgical treatment may be associated with a lower risk of preterm labor, without increased risks of abortion and cesarean section.

## Introduction

Pregnancy complicated by acute appendicitis (AA) is a severe and frequent indication for surgery during pregnancy, with an incidence of 0.05–0.13 per 100 pregnancies [1]. Compared with pregnancies without AA, pregnancies affected by AA tended to have poorer outcomes, such as increased risks of abortion, preterm labor, and cesarean section [2]. In clinical practice, three treatment options are available for pregnancy complicated with AA: open appendectomy (OA), laparoscopic appendectomy (LA), and non-surgical treatment (NST). If the pathological condition of AA is not severe, or the patient’s physical condition is temporarily unsuitable for surgical treatment, NST, a conservative treatment method (e.g., with antibacterial and/or anti-inflammatory drugs) is often chosen. In Korea, approximately 25% of pregnancies affected by uncomplicated AA are treated conservatively [3], compared to 63% in China [4]. In contrast, for complicated AA in pregnancy, OA and LA are the two most frequent choices. The World Society of Emergency Surgery suggests LA be preferred to OA for pregnant patients in the presence of surgery indications [5], because it has the advantages of less pain, shorter hospital stay, and lower infection risk at the surgical site [6]. Some reports have suggested that LA is associated with higher risk of fetal loss as compared with OA [5–11]. Therefore, OA remains widely used by surgeons for pregnant patients affected by AA. However, studies that found increased risk of fetal loss usually had small and highly selective samples [5–11].

Determinants for outcomes of pregnancies affected by AA could be multifactorial. Although individual-level determinants of pregnancy outcomes such as demographic and obstetric complications for AA have been studied [12], hospital-level determinants have not been investigated. Hospital-level factors have been found to be associated with outcomes in other health conditions such as mortality in patients with head and neck cancers [13] and in cirrhosis patients [14]. We therefore carried out a retrospective cohort study of pregnancies affected by AA based on a large patient population from the United States to further explore the association between treatment method and pregnancy outcomes. The specific objectives of this study were: (1) to assess the secular trends of treatment methods for pregnancies affected by AA; (2) to examine pregnancy outcomes after OA, LA, and NST for pregnancies affected by AA; (3) to investigate associations of patient- and hospital-level characteristics with pregnancy outcomes based on multilevel hierarchal regression analysis.

## Methods

### Study design and data source

This retrospective cohort study involved pregnancies affected by AA and treated in 632 hospitals in the United States between 2000 and 2016 and recorded by Cerner Health Facts Database, which is a large data repository containing more than 100 million medical information entries [15]. The Cerner Health Facts Database uses automated electronic health record systems to capture encounter-level patient data. Data include medical history, hospital procedures, diagnostic data, laboratory orders and results, prescription information and diagnoses codes.

### Study population

The study population included women 15–49 years of age and who developed AA during pregnancy. AA during pregnancy was defined by ICD-9 and ICD-10 codes, Diagnosis Related Group (DRG) codes, and procedure codes (codes are shown in S1 Table). Exclusion criteria included missing record identifiers, duplicate records, multiple gestation pregnancy, and patients who underwent appendectomy for causes unrelated to acute appendicitis.

### Outcome measures

The outcome measures of this study were abortion (ICD-9 codes: 632, 634.x, 637.x, 640.x, 656.4x, and ICD-10 codes: O03, O20.0, O31.1), preterm labor (ICD-9 codes: 644.2, 644.20, 644.21, and ICD-10 codes: O60, P05.1, P07.3) and cesarean section (ICD-9-CM code: 654.2, V30.01, V33.01, and ICD-10 codes: O82, Z38.01).

### Determinants of adverse pregnancy outcomes

The method of treatment of AA in pregnancy was the most important individual-level determinant of adverse pregnancy outcomes, which were divided into three groups: OA, LA, and NST. ICD-9 procedure codes and the Current Procedural Terminology Coding System, Fourth Edition (CPT-4) codes used to define the three treatments. OA was identified with specific ICD-9 procedure codes (47.0, 47.09, 47.19) and CPT-4 codes (9965, 9966, 9967). LA was identified with specific ICD-9 procedure codes (47.01, 47.11) and CPT-4 codes (9968, 10894). All remaining cases (without specific surgical procedure codes) were classified as NST.

Other individual-level determinants of adverse pregnancy outcomes considered in this study included age, race/ethnicity, residence (urban/rural), and pregnancy complications. Pregnancy complications that were considered as potentially associated with adverse pregnancy outcomes included hypertension disorders in pregnancy (ICD-9 codes: 642.3, 642.3x, 642.9, 642.9x, and ICD-10 codes:O11, O11.x, O13, O13.x), diabetes complicating pregnancy (ICD-9 codes:648.8x, and ICD-10 codes: O24.435, O24.415, O24.425, O24.4 O24.4x), hyperlipidemia (ICD-9 codes: 272.2, 272.4 and ICD-10 codes: E78.2, E78.4, E78.5), obesity (ICD-9 codes: 278, 278.0, 278.00, 278.01, 278.03, 649.1x, V77.8 and ICD-10 codes: O99.21x, E66.x (excluding E66.3) and anemia (codes are shown in S2 Table).

Hospital-level determinants considered in this study included geographic area of hospital location and number of hospital beds. All determinants included in the analysis were considered to be independently associated with pregnancy outcomes.

### Statistical analysis

We first described distribution of treatment methods of AA in pregnancy and other study variables. We then applied univariate logistic regression models to examine the association of treatment methods and other independent variables with pregnancy outcomes. Finally, we used multivariable hierarchical logistic regressions to analyze the association of treatment methods and other individual- and hospital-level factors with abortion, preterm labor, and cesarean delivery respectively, with the odds ratio (OR) and 95% confidence interval (CI) as the association measure. A full-adjusted model was fit including all independent variables, with individual-level variables randomly intercepting with corresponding hospital-level variables. All statistical analyses were performed using SAS statistical software (Version 9.4 for Windows; SAS Institute, Cary, North Carolina, USA) and HLM7.0.

### Informed consent

This study was approved by the Ottawa Health Science Network Research Ethics Board (OHSN-REB). The Cerner Health Facts data is compliant with the Health Insurance Portability and Accountability Act (HIPAA). Informed patient consent and ethical review were waived.

## Results

### Baseline demographics and clinical characteristics of the study population

A total of 10,271 cases of acute appendicitis during pregnancy were identified from Cerner Health Facts data between 2000 and 2016. Of them, 1,403 (13.7%) were treated by OA, 5,872 (57.2%) by LA, and 2,996 (29.2%) by NST. Only 8 women had acute appendicitis during pregnancy twice, all of whom were treated with NST. In all pregnant women with acute appendicitis, abortion occurred in 507 (4.9%), of which 152 (1.5%) and 162 (1.6%) underwent preterm labor and cesarean section, respectively.

**Fig 1:**
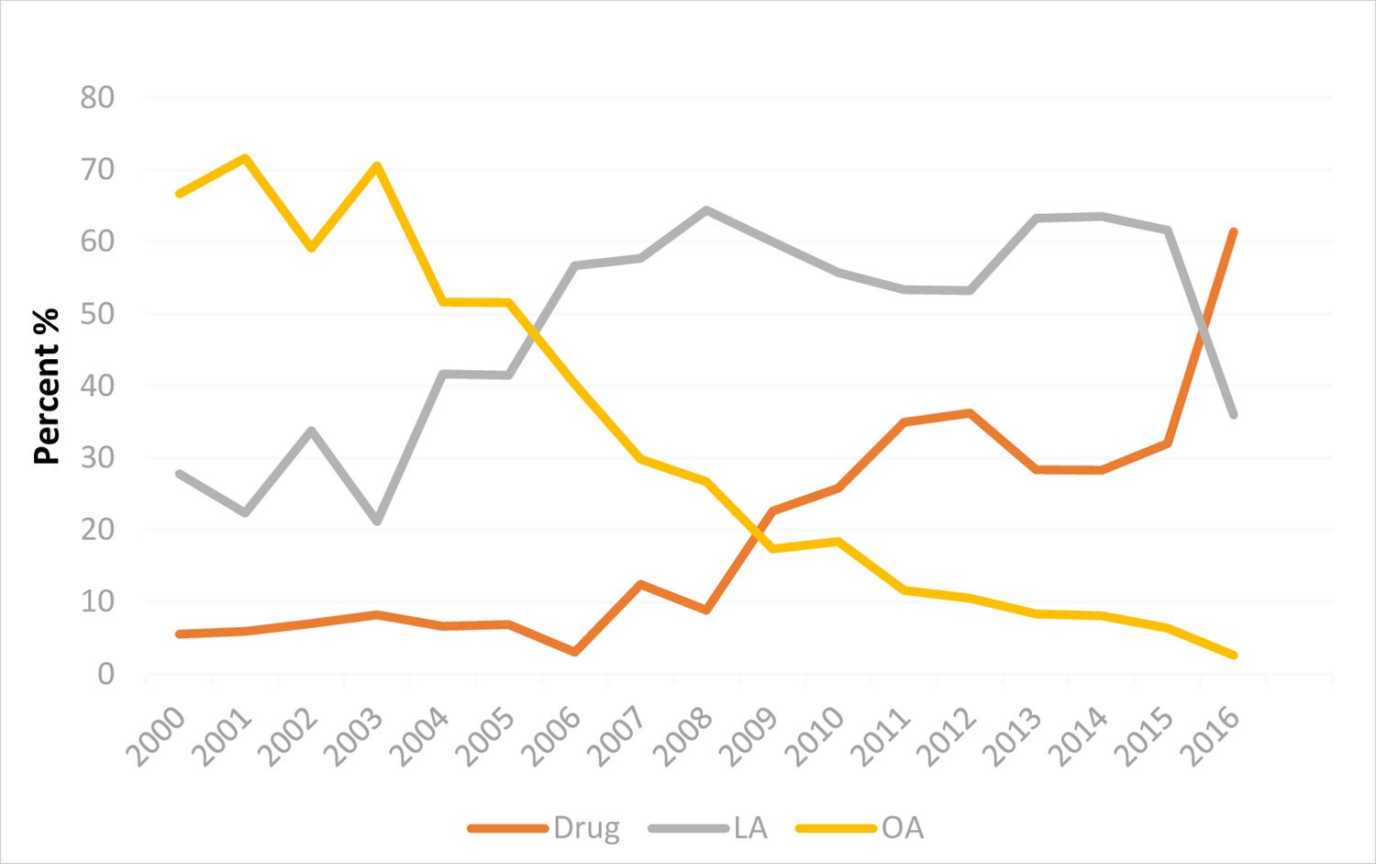
Changes in the constituent ratios of the three treatment methods for acute appendicitis in pregnancy [drug, pharmaceutical treatment; LA, laparoscopic appendectomy; OA, open appendectomy].

Fig 1 shows the temporal trends of the three treatment methods. Surgical therapy showed an overall downward trend from 2000 to 2016, whereas conservative method was relatively stable from 2000 to 2007, then demonstrated an upward trend between 2007 and 2012, followed by a steady decline after 2013 and then a sharp increase in 2016. LA showed an upward trend before 2008, remained stable from 2008 to 2013, then decreased from 2014 to 2016 (Fig 1). The recent overall decrease in LA was attributable primarily to the increase in NST. For cases treated by surgery, most were treated by LA rather than OA.

Table 1 summarizes the individual characteristics of the 10,271 patients along with the corresponding hospital-level characteristics. The mean age of the patients was 29.1 years and most of these patients (71%) were Caucasians. Eighty-four percent of these patients were treated in urban hospitals, 28.9% in hospitals located in the northeast United States, and 41.8% were treated in hospitals with a bed size of 300 or greater.

**Table 1:** Individual- and hospital-level characteristics and treatment of AA in pregnant women (N = 10,271).

Variable	Number of Patients (%)
**Individual-level characteristics**	
** Age in years**	
<20	1,543 (15.0)
20–24	2,240 (21.8)
25–29	2,171 (21.1)
30–34	1,807 (17.6)
35–49	2,510 (24.4)
** Race/ethnicity**	
African America	1,056 (10.3)
Caucasian	7,293 (71.0)
Others	1,922 (18.7)
** Comorbidity**	
Diabetes	
Yes	739 (7.2)
No	9,532 (92.8)
Hypertension	
Yes	1,089 (10.6)
No	9,182 (89.4)
Anemia	
Yes	1,090 (10.6)
No	9,181 (89.4)
Hyperlipidemia	
Yes	374 (3.6)
No	9,897 (96.4)
Obesity	
Yes	1,076 (10.5)
No	9,195 (89.5)
**Hospital-level characteristics**	
Area type	
Rural	1,628 (15.9)
Urban	8.643 (84.1)
Region	
Midwest	2,298 (22.4)
Northeast	2,973 (28.9)
South	2,926 (28.5)
West	2,074 (20.2)
Number of beds	
≤99	1,990 (19.3)
100–299	3,997 (38.9)
≥300	4,284 (41.8)
**AA treatment method**	
Open appendectomy	1,403 (13.7)
Laparoscopic appendectomy	5,872 (57.2)
Non-surgical treatment	2,996 (29.2)

### Determinants of abortion

Table 2 presents results of analysis of determinants of abortion. No difference in risk of abortion among the three groups with different treatment methods was observed. Individual-level factors associated with increased risk of abortion were maternal age and anemia. For hospital-level determinants, a bed size of 99 or less was associated with decreased risk of abortion.

**Table 2:** Multilevel hierarchical logistic regression analysis of the potential factors for abortion.

Variable	No. (%)	OR (95% CI)	*P*	aOR (95% Cl)*	*P*
**Individual-level characteristics**					
**Social background information**					
**Age (years)**					
<20	56 (11.1)	1.0 (0.7–1.5)	0.837	1.0 (0.7–1.5)	0.949
20–24	132 (26.0)	1.7 (1.3–2.3)	0.000	1.7 (1.2–2.4)	0.005
25–29	131 (25.8)	1.8 (1.3–2.3)	0.000	1.8 (1.2–2.5)	0.002
30–34	100 (19.7)	1.6 (1.2–2.2)	0.001	1.7 (1.2–2.3)	0.004
35–49	88 (17.4)	ref		ref	
**Race/ethnicity**					
African American	68 (13.4)	0.6 (0.5–0.8)	0.000	1.2 (0.9–1.7)	0.213
Other	125 (24.7)	1 (0.7–1.3)	0.946	1.2 (1.0–1.6)	0.052
Caucasian	314 (61.9)	ref		ref	
**Comorbidity**					
** Diabetes**					
Yes	59 (11.6)	1.8 (1.3–2.3)	0.000	1.3 (0.9–1.9)	0.103
No	448 (88.4)	ref		ref	
** Hypertension**					
Yes	64 (12.6)	1.2 (0.9–1.6)	0.13	1.1 (0.8–1.5)	0.667
No	443 (87.4)	ref		ref	
** Anemia**					
Yes	88 (17.4)	1.8 (1.4–2.3)	0.000	1.5 (1.1–2.0)	0.004
No	419 (82.6)	ref		ref	
** Hyperlipidemia**					
Yes	30 (5.9)	1.7 (1.2–2.5)	0.006	1.2 (0.7–2.3)	0.492
No	477 (94.1)	ref		ref	
** Obesity**					
Yes	74 (14.6)	1.5 (1.2–1.9)	0.002	1.2 (0.9–1.6)	0.168
No	433 (85.4)	ref		ref	
**Hospital-level characteristics**					
**Characteristics of hospitals**					
**Area**					
Urban	407 (80.3)	1.3 (1.1–1.7)	0.015	1.0 (0.7–1.4)	0.918
Rural	100 (19.7)	ref		ref	
**Geographical location**					
Midwest	85 (16.8)	0.6 (0.5–0.8)	0.000	0.7 (0.5–1.1)	0.126
Northeast	165 (32.5)	0.9 (0.7–1.2)	0.474	1.1 (0.7–1.5)	0.633
South	132 (26.0)	0.7 (0.6–0.9)	0.017	0.8 (0.6–1.2)	0.314
West	125 (24.7)	ref		ref	
**Bed size**					
≤99	58 (11.4)	0.5 (0.4–0.7)	0.000	0.6 (0.4–1.0)	0.044
100–299	200 (39.4)	0.9 (0.7–1.0)	0.105	0.9 (0.7–1.2)	0.496
≥300	249 (49.2)	ref		ref	
**Treatment method for AA**					
Drug	151 (29.8)	0.9 (0.7–1.2)	0.532	1.0 (0.7–1.4)	0.855
Laparoscopic appendectomy	279 (55.0)	0.9 (0.7–1.1)	0.251	0.9 (0.7–1.2)	0.568
Open appendectomy	77 (15.2)	ref		ref	

* All independent variables in the table were adjusted each other simultaneously.

### Determinants of preterm labor

Table 3 presents results of analysis of determinants of preterm labor. Compared with OA, both LA (aOR, 0.6; 95% CI, 0.4–0.9) and NST (aOR, 0.4; 95% CI, 0.3–0.7) were associated with a reduced risk of preterm labor. Other individual-level factors associated with increased risk of preterm labor were maternal age, hypertension disorders in pregnancy, and anemia (Table 3). For hospital-level variables, there were no difference in the risk of preterm labor (Table 3).

**Table 3:** Multilevel hierarchical logistic regression analysis of the potential factors for preterm labor.

Variable	No. (%)	OR (95% CI)	*P*	aOR (95% Cl)*	*P*
**Individual-level characteristics**					
**Social background information**					
**Age-y**					
<20	12 (7.9)	0.9 (0.5–1.9)	0.839	2.2 (1.0–4.7)	0.042
20–24	46 (30.3)	2.5 (1.5–4.2)	0.001	4.1 (2.5–6.7)	0.000
25–29	39 (25.6)	2.2 (1.3–3.7)	0.004	2.9 (1.7–4.9)	0.000
30–34	34 (22.4)	2.3 (1.37–3.9)	0.003	2.6 (1.5–4.4)	0.001
35–49	21 (13.8)	ref		ref	
**Race/ethnicity**					
African American	16 (10.5)	1.2 (0.8–1.8)	0.475	1.3 (0.7–2.3)	0.441
Other	25 (16.5)	1.2 (0.6–2.2)	0.631	0.9 (0.6–1.4)	0.735
Caucasian	111 (73.0)	ref		ref	
**Comorbidity**					
** Diabetes**					
Yes	16 (10.5)	1.5 (0.9–2.6)	0.112	1.3 (0.8–2.1)	0.260
No	136 (89.5)	ref		ref	
** Hypertension**					
Yes	34 (22.4)	2.5 (1.7–3.6)	< .001	2.7 (1.9–3.8)	0.000
No	118 (77.6)	ref		ref	
** Anemia**					
Yes	45 (29.6)	3.7 (2.6–5.2)	< .001	3.3 (2.4–4.7)	0.000
No	107 (70.4)	ref		ref	
** Hyperlipidemia**					
Yes	4 (2.6)	0.7 (0.3–1.9)	0.505	0.4 (0.1–1.4)	0.151
No	148 (97.4)	ref		ref	
** Obesity**					
Yes	20 (13.2)	1.3 (0.8–2.1)	0.278	1.0 (0.6–1.8)	0.862
No	132 (86.8)	ref		ref	
**Hospital-level characteristics**					
**Area**					
Urban	119 (78.3)	0.7 (0.5–1.0) (0.457–0.996)	0.048	0.7 (0.5–1.1)	0.169
Rural	33(21.7)	ref		ref	
**Geological location**					
Midwest	36 (23.7)	1.3 (0.8–2.2)	0.311	1.4 (0.8–2.6)	0.252
Northeast	63 (41.5)	1.8 (1.1–2.8)	0.016	1.7 (0.9–3.1)	0.093
South	28 (18.4)	0.8 (0.5–1.4)	0.399	0.7 (0.4–1.4)	0.352
West	25 (16.4)	ref		ref	
**Bed size**					
≤99	26 (17.1)	0.7 (0.5–1.1)	0.175	1.0 (0.6–1.7)	0.992
100–299	50 (32.9)	0. 7(0.5–1.0)	0.053	0.8 (0.5–1.3)	0.37
≥300	76 (50.0)	ref		ref	
**Treatment method for AA**					
Drug	31 (20.4)	0.3 (0.2–0.6)	< .001	0.4 (0.3–0.7)	0.002
Laparoscopic appendectomy	80 (52.6)	0.5 (0.3–0.7)	< .001	0.6 (0.4–0.9)	0.009
Open appendectomy	41 (27.0)	ref		ref	

* All independent variables in the table were adjusted each other simultaneously.

### Determinants of cesarean section

Table 4 presents results of analysis of determinants of cesarean section. No difference in the risk of cesarean section with different treatment methods among the three groups was observed (Table 4). Individual-level determinants associated with increased risk of cesarean section were maternal age and anemia (Table 4). No hospital-level determinant was associated with cesarean section (Table 4).

**Table 4:** Multilevel hierarchical logistic regression analysis of the potential factors for cesarean section.

Variable	No. (%)	OR (95% CI)	*P*	aOR (95% Cl)*	*P*
**Individual-level characteristics**					
**Social background information**					
**Age, y**					
<20	16 (9.9)	0.8 (0.4–1.5)	0.497	1.4 (0.7–2.9)	0.297
20–24	35 (21.6)	1.2 (0.8–2.0)	0.402	1.7 (1.0–2.9)	0.036
25–29	39 (24.1)	1.4 (0.9–2.3)	0.147	1.7 (1.0–2.8)	0.047
30–34	40 (24.7)	1.8 (1.1–2.8)	0.019	1.9 (1.1–3.3)	0.018
35–49	32 (19.7)	ref		ref	
**Race/ethnicity**					
African American	13 (8.0)	0.7 (0.5–1.1)	0.109	0.9 (0.5–1.5)	0.71
Other	39 (24.1)	0.6 (0.3–1.1)	0.115	1.2 (0.8–1.9)	0.279
Caucasian	110 (67.9)	ref		ref	
**Comorbidity**					
** Diabetes**					
Yes	23 (14.2)	2.2 (1.4–3.4)	0.001	1.6 (1.0–2.9)	0.073
No	139 (85.8)	ref		ref	
** Hypertension**					
Yes	28 (17.3)	1.8 (1.2–2.7)	0.006	1.4 (0.8–2.3)	0.242
No	134 (82.7)	ref		ref	
** Anemia**					
Yes	51 (31.5)	4 (2.9–5.6)	< .001	3.7 (2.5–5.7)	<.001
No	111 (68.5)	ref		ref	
** Hyperlipidemia**					
Yes	10 (6.2)	1.8 (0.9–3.4)	0.087	0.9 (0.4–2.0)	0.85
No	152 (93.8)	ref		ref	
** Obesity**					
Yes	28 (17.3)	1.8 (1.2–2.7)	0.005	1.3 (0.8–2.1)	0.233
No	134 (82.7)	ref		ref	
**Hospitals-level characteristics**					
**Characteristics of hospitals**					
**Area**					
Urban	131 (80.9)	1.3 (0.9–1.9)	0.249	0.8 (0.4–1.4)	0.37
Rural	31 (19.1)	ref		ref	
**Geographic location**					
Midwest	36 (22.2)	1.0 (0.6–1.7)	0.847	1.1 (0.6–2.3)	0.721
Northeast	63 (38.9)	1.4 (0.9–2.2)	0.108	1.5 (0.8–2.9)	0.227
South	32 (19.8)	0.7 (0.4–1.2)	0.212	0.7 (0.4–1.3)	0.284
West	31 (19.1)	ref		ref	
**Bed size**					
≤99	31 (19.1)	0.9 (0.6–1.4)	0.722	1.5 (0.8–2.8)	0.257
100–299	59 (36.4)	0.9 (0.6–1.2)	0.456	1.2 (0.7–2.2)	0.477
≥300	72 (44.5)	ref		ref	
**Treatment method for AA**					
Drug	52 (32.1)	1.1 (0.7–1.8)	0.687	1.0 (0.5–1.7)	0.862
Laparoscopic appendectomy	88 (54.3)	1.0 (0.6–1.5)	0.848	1.0 (0.6–1.7)	0.968
Open appendectomy	22 (13.6)	ref		ref	

* All independent variables in the table were adjusted for simultaneously.

## Discussion

### Main findings

Our retrospective cohort study of a large number of pregnancies covering all regions of the United States found an overall downward trend in use of surgical therapy, along with an upward trend in conservative treatment for pregnancies affected by AA. LA showed an upward trend prior to 2008, plateaued from 2008 to 2013, then decreased notably between 2014 to 2016. Compared OA, LA and NST were associated with a lower risk of preterm labor. On the other hand, no difference in the risks of abortion and cesarean section among the three treatment methods was observed. Other important individual-level determinants of adverse pregnancy outcomes were maternal age, hypertension disorders in pregnancy, and anemia. The only hospital-level determinant of adverse pregnancy outcomes was bed size.

### Strength and limitations

Our study has several strengthens. First, it was based on a large sample from 632 hospitals in the United States. To our knowledge, this is the largest study in the field. Second, we have used multilevel regression analysis, which is appropriate for data measured at different levels. If the hierarchical nature of the data is ignored, a large amount of information may be lost and the chance of a type I error (false positive) may be increased [16]. Third, the data spanned a follow-up period of more than a decade, which allowed an analysis of trends in treatment methods for AA in pregnancy, which will be of interest to health care providers considering treatment options.

Limitations of this study should be acknowledged. First, as accurate information on gestational age was not available, it is difficult to determine at which stage of pregnancy treatment for AA was performed. Technically it is not possible to perform LA after the 2^nd^ trimester because the uterus is too large to safely perform laparoscopic surgery. Moreover, because of the lack of information on gestational age, we could not perform an analysis of preterm birth. Instead, we have used ICD codes for preterm labor as a proxy for preterm birth. Second, we were not able to control for the severity of illness. Those cases that needed surgical intervention were systemically much more ill, raising the possibility of confounding by indication for which we were unable to adjust. Third, although a number of hospital-level and individual-level determinants were adjusted for simultaneously, residual confounding caused by unmeasured variables may still exist. Fourth, administrative data of the type analyzed here may be subject to a certain level of coding errors [17], although the Cerner data are considered to be of high quality [18]. Lastly, the data presented in our study was extracted from a electronic health records database in the United States. It remains unclear whether our findings could be generalized into other counties.

### Interpretation and implications

Our study used multilevel modeling, which is superior to conventional regression modeling in analyzing hierarchical data. When conventional logistic regression was used to analyze associations between determinants and outcomes in pregnancies affected by AA [2, 19, 20], it was assumed that individual patients were independent. However, with hierarchical data, disease severity may vary among hospitals in which the patients were treated. Our study found for the first time that hospital size was significantly associated with abortion, which further emphasizes the importance of adjusting for hospital-level determinants in the analysis of individual-level determinants, and vice-versa. The underlying reasons why smaller hospitals had higher abortion rates is not entirely clear. Bed size may be related to surgical volume, and previous studies suggested that poor surgical outcomes observed in low volume hospitals may be caused by lack of surgical skills in low volume hospitals [21].

Our study showed that NST was associated with lower risk of preterm labor by 60% as compared with OA, but no difference in abortion and cesarean section between NST and OA treatments was seen. Although some previous studies suggested that conservative treatment of AA in pregnant women achieved satisfactory pregnancy outcomes, these patients normally had relatively mild conditions [4]. The World Society of Emergency Surgery guidelines note that no evidence supports superior performance of conservative treatment in pregnancy outcomes, as compared with surgical treatments [5].

Previous studies have assessed the association of laparoscopy with adverse pregnancy outcomes, particularly with fetal loss, preterm birth, and cesarean section. While a significantly lower risk of preterm birth was found in patients treated by LA than OA [22], other studies found no difference in preterm birth between the two surgical approaches [2, 3, 4], and few studies found that LA was associated with increased risk of fetal loss [6, 22–24]. A case–control study reported that LA during pregnancy was associated with an increased rate of cesarean delivery [12], while other studies found that the risk of cesarean delivery for LA and OA treatments was comparable [6, 25]. Our study showed that compared with OA, LA was associated with reduced risk of preterm labor by 40%, whereas no difference in the risk of abortion or cesarean section between LA and OA was found. Our study involved more patients than the total number of patients summarized in a previous meta-analysis [6], and used multi-level regression to adjust for individual- and hospital-level determinants simultaneously. These differences may explain the discrepancies between our study and previous studies. Our study results support the current recommendations of the Society of the American Gastrointestinal and Endoscopic Surgeons and the World Society of Emergency Surgery guidelines that LA is preferred to OA for pregnancies affected by AA in the presence of surgery indications [5, 26]. The recent overall decrease in LA was driven mostly by the increase in NST. For cases treated by surgery, most were treated by LA instead of OA, suggesting that surgeons in the United States have followed the international and United States guidelines for pregnancies affected by AA.

## Conclusion

Based on a large sample of pregnancies from all regions in the United States between 2000 and 2016, that compared with OA, LA was associated with lower risk of preterm labor, without increased risks of abortion and cesarean section.

## Supporting information

S1 Table Diagnosis codes for acute appendicitis during pregnancy. (DOCX)

S2 Table Diagnosis codes for anemia. (DOCX)
