# Supplementary material for: Association of treatments for acute appendicitis with pregnancy outcomes in the United States from 2000 to 2016: Results from a multi-level analysis
Source: PLoS One. 2021 Dec 13;16(12):e0260991. doi: 10.1371/journal.pone.0260991 (PMC8668090; doi:10.1371/journal.pone.0260991)
Supplement: S1 Table — (DOCX) [file pone.0260991.s001.docx]

**Supplement Table 1.** Diagnosis Codes for acute appendicitis during Pregnancy

| **Outcome** | **Category** | **Codes** |
| --- | --- | --- |
| Acute appendicitis | ICD-9 codes | 540.x |
|  | ICD-10 codes | K35，K35.x |
| Pregnancy | ICD-9 codes | 650，V27，V30，V31，V32，V33，V34，V35，V36，V37 |
|  | ICD-10 codes | Z34.8, Z34.9 |
|  | DRG codes | 370，371，372，373，374，375 |
|  | PROCEDURE codes | 72.5，72.39，72.21，72.53，72.71，72.3，74.1，73.6，74.2，72.6，72.8，73.59，72.79，72.51，74.4，72.29，72.31，72.9，72.4，74.9，72.1，73.22，72.7，72.54，74.0，72.2，72.0 |
