# Supplementary material for: Association of treatments for acute appendicitis with pregnancy outcomes in the United States from 2000 to 2016: Results from a multi-level analysis
Source: PLoS One. 2021 Dec 13;16(12):e0260991. doi: 10.1371/journal.pone.0260991 (PMC8668090; doi:10.1371/journal.pone.0260991)
Supplement: S2 Table — (DOCX) [file pone.0260991.s002.docx]

**Supplement Table 2.** Diagnosis Codes for Anemia

| **Coding System** | **Codes** |
| --- | --- |
| ICD-9 codes | 280.x，281.x，282，282.2，282.3，282.8，282.9，283，283.0，283.1，283.10，283.19，283.9，284，284.0，284.09，284.8，284.89，284.9，285，285.0，285.2，285.21，285.22，285.29，285.3，285.8，285.9，285.1，648.2x，776.5，773.5，774.0，776.6，V78.0，V78.1 |
| ICD-10 codes | O90.81，P61.2，P61.3，P61.4，O99.0，O99.01，O99.011，O99.012，O99.013，O99.019，O99.02，O99.03 |
